# Synthesis and structural studies of a new complex of *catena*-poly[*p*-anisidinium [[diiodidobismu­thate(III)]-di-μ-iodido] dihydrate]

**DOI:** 10.1107/S2056989015019489

**Published:** 2015-10-24

**Authors:** Mohamed El Mehdi Touati, S. Elleuch, Habib Boughzala

**Affiliations:** a Laboratoire de Matériaux et Cristallochimie, Faculté des Sciences de Tunis, Université de Tunis El Manar, 2092 Manar II Tunis, Tunisia; b Laboratoire de Physique appliquée, Faculté des Sciences de Sfax, 3018 BP 802, Tunisia

**Keywords:** crystal structure, bis­muth iodide, [BiI_6_] octa­hedron, hydrogen bonds, *p*-anisidinium, hybrid complex

## Abstract

A new organic–inorganic hybrid material, {(C_7_H_10_NO)[BiI_4_]·2H_2_O}_*n*_, has been synthesized by slow evaporation of an aqueous solution at room temperature. The anionic sublattice of the crystal is built up by [BiI_6_] octa­hedra sharing edges. The resulting zigzag chains extend along the *a*-axis direction and are arranged in a distorted hexagonal rod packing. The *p*-anisidinium cations and the water mol­ecules are located in the voids of the anionic sublattice. The cations are linked to each other through N—H⋯O hydrogen bonds with the water mol­ecules, and also through weaker N—H⋯I inter­actions to the anionic inorganic layers.

## Abstract 2

The anionic network of the new hybrid compound (C_7_H_10_NO)[BiI_4_]·2H_2_O is built up by edge-sharing [BiI_6_] octa­hedra leading to a one-dimensional structural topology. Hydrogen bonds ensure the crystal cohesion by connecting the alternating organic–inorganic layers and building a three-dimensional framework.

## Chemical context   

Previous X-ray structural studies showed that halogenidobismuthate(III) complexes may contain an array of variously self-organized halobismuthate anions since different polynuclear species can be formed through oligomerization by halide bridging (Bowmaker *et al.*, 1998 ▸; Benetollo *et al.*, 1998 ▸; Alonzo *et al.*, 1999 ▸).

In general, the coordination sphere of bis­muth appears to be dominated by an hexa­coordination tendency with polybismuthate species arising from corner-, edge- or face-sharing [Bi*X*_6_] distorted octa­hedra. If the anionic sublattice dimensionality is clearly determined by the counter-cations, the effects of their most evident properties such as charge, size and shape are not predictable. Organic cations resulting from protonated nitro­gen functionalities may provide a rich family of salts where the factors cited above could be varied rationally. In addition, since the important contribution to the lattice stabilization in the crystalline state is due to hydrogen-bonding inter­actions, it should be possible to influence the bis­muth coordination geometry by changing the number and orientation of the hydrogen-bond donor sites of the cations. In an effort to increase the size of the [Bi*X*_6_] octahedra, iodine was used in the chemical synthesis.
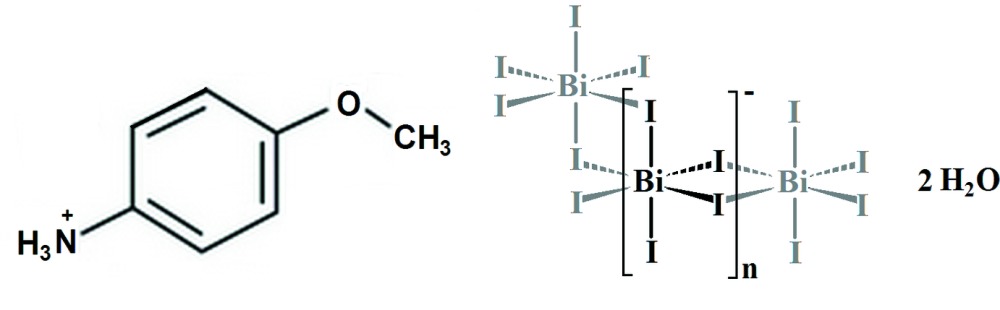


## Structural commentary   

**Figure 1:**
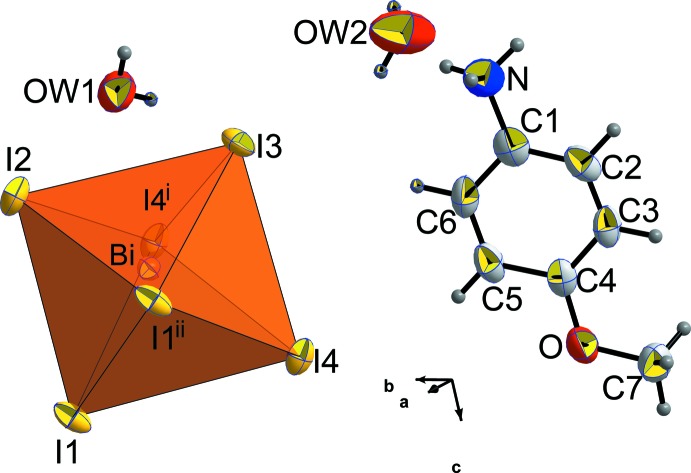
Representation of the structural units of (C_7_H_10_NO)[BiI_4_]·2H_2_O, with 50% probability displacement ellipsoids. [Symmetry codes: (i) 1 − *x*, 1 − *y*, 1 − *z*; (ii) 2 − *x*, 1 − *y*, 1 − *z*.]

**Figure 2:**
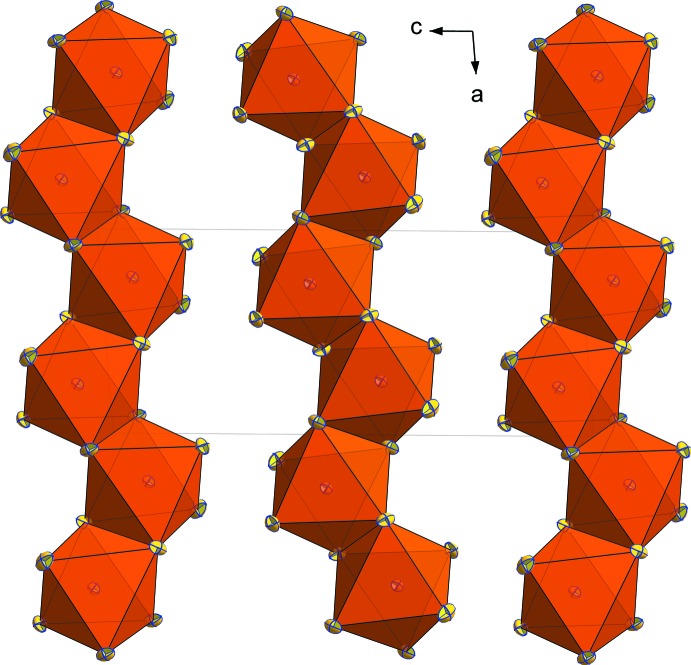
View of the anionic framework in the structure of (C_7_H_10_NO)[BiI_4_]·2H_2_O, showing the zigzag chains running along the *a-*axis direction.

The principal building blocks of the title compound are octahedral iodidobismuthate [BiI_6_] units, *p*-anisidinium cations and two water mol­ecules (Fig. 1 ▸). The anionic sublattice of the crystal is built of one-dimensional zigzag chains extending along the *a*-axis direction and composed of [BiI_6_] octa­hedra sharing edges as shown in Fig. 2 ▸. The one-dimensional secondary building unit (SBU) topology observed in the described structure is one of the most common and stable ones (Billing & Lemmerer, 2006 ▸) in bis­muth halide hybrids. The shortest Bi—Bi distance [4.590 (1) Å] observed is in agreement with homologous structures having the same one-dimensional topology. The octa­hedral bis­muth coordination is almost regular, proving the stereochemical inactivity of the Bi^3+^ 6*s* ^2^ electron lone pair. Furthermore, among the six octa­hedral vertices, two are monocoordinated with short bond lengths (I2 and I3), while the four others (I4, I1 and symmetry-related atoms) are bicoordinated exhibiting long bond lengths (Table 1 ▸).

**Figure 3:**
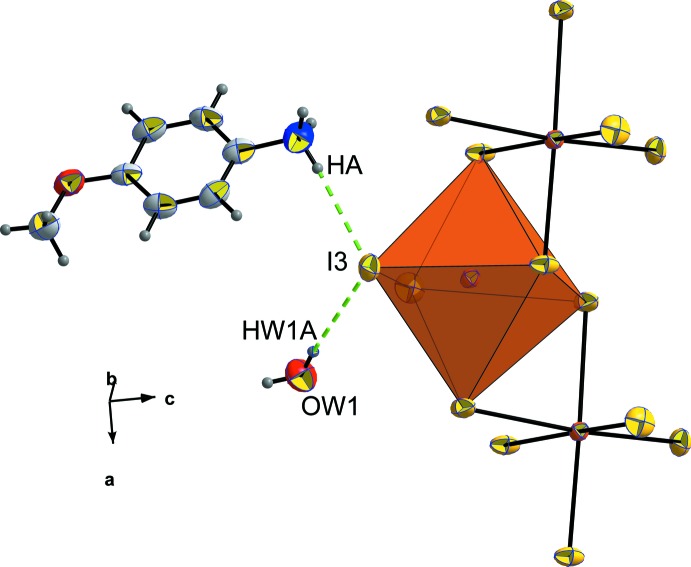
The environment of the [BiI_6_] octa­hedron in the structure of (C_7_H_10_NO)[BiI_4_]·2H_2_O. [Symmetry codes: (i) −*x* + 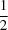, *y* − 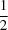, −*z* + 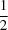; (ii) −*x* + 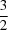, *y* − 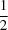, −*z* + 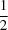.]

In Fig. 3 ▸, it can be seen that each [BiI_6_] octa­hedron is linked to one *p*-anisidinium cation and a water mol­ecule O*W*1 *via* I3⋯H*A*—N and I3⋯H*W*1*A*—O*W*1 hydrogen bonds.

**Figure 4:**
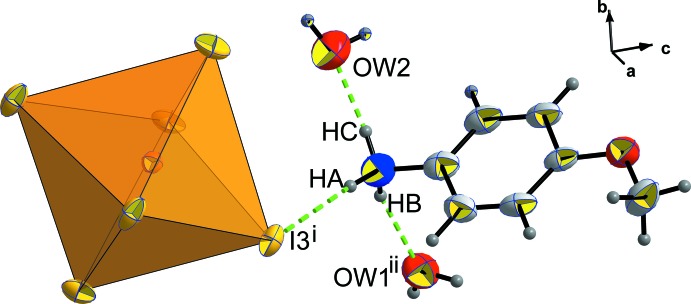
The environment of the *p*-anisidinium cation in the structure of (C_7_H_10_NO)[BiI_4_]·2H_2_O. [Symmetry codes: (i) −*x* + 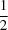, *y* − 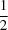, −*z* + 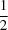; (ii) 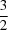 − *x*, −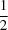 + *y*, 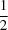 − *z*.]

The *p*-anisidinium cation is adopting a quite planar configuration characterized by a slight r.m.s. deviation of 0.020 (9) Å. Each *p*-anisidinium cation inter­acts with one [BiI_6_] octa­hedron *via* N—H*A*⋯I3^i^ (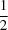 − *x*, −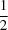 + *y*, 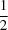 − *z*) , with two water mol­ecules by N—H*B*⋯O*W*1^ii^ (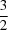 − *x*, −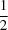 + *y*, 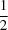 − *z*) and N—H*C*⋯O*W*2 hydrogen bonds (Table 2 ▸), as shown in Fig. 4 ▸.

## Supra­molecular features   

**Figure 5:**
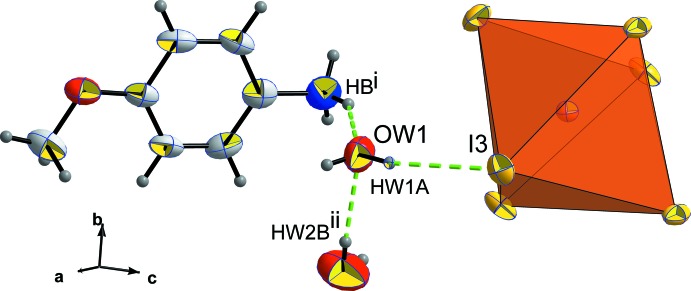
The environment of the O*W*1 water mol­ecule. [Symmetry codes: (i) 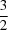 − *x*, 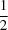 + *y*, 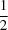 − *z*; (ii) *x* + 1, *y*, *z*.]

**Figure 6:**
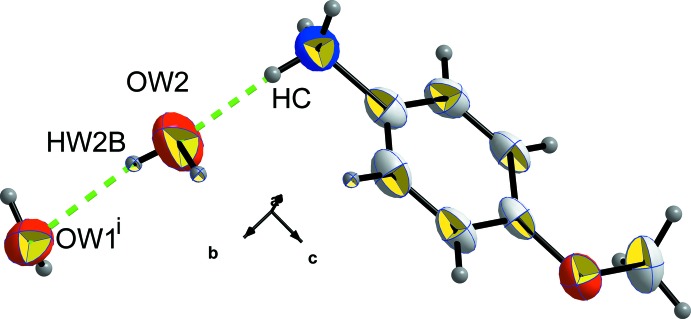
The environment of the O*W*2 water mol­ecule. [Symmetry code: (i) −1 + *x*, *y*, *z.*]

**Figure 7:**
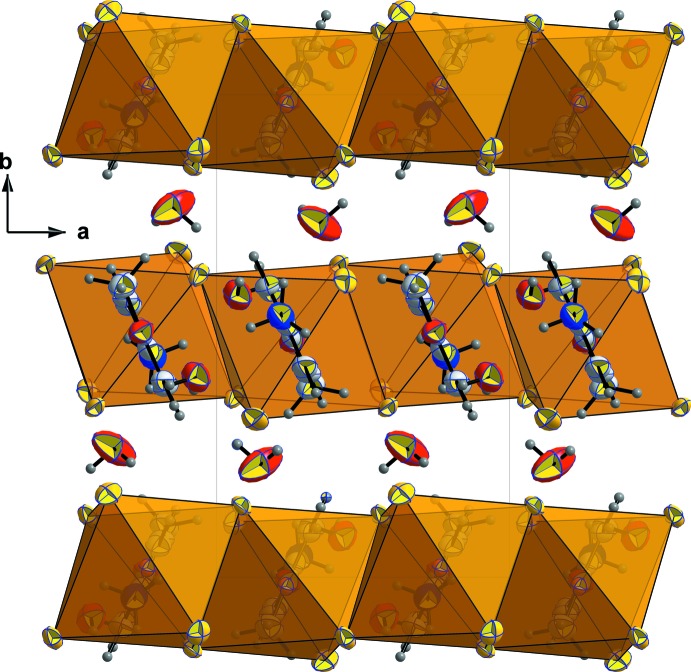
The mol­ecular stacking along the *b* axis, showing the empty inter­layer space where the O*W*2 water mol­ecules are located.

The role of the water mol­ecules is crucial in the crystal cohesion. In fact, O*W*1 is engaged in three hydrogen bonds to one organic cation, one [BiI_6_] octa­hedra and one water mol­ecule *via* O*W*1⋯H*B* ^i^—N^i^, O*W*1—H*W*1*A*⋯I3 and O*W*1⋯H*W*2*B* ^ii^—O*W*2^ii^, respectively, as shown in Fig. 5 ▸ [symmetry codes: (i) 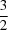 − *x*, 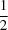 + *y*, 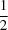 − *z*; (ii) *x* + 1, *y*, *z*). The second water mol­ecule O*W*2 is linked to O*W*1 by O*W*2—H*W*2*B*⋯O*W*1(−1 + *x*, *y*, *z*) and to the *p*-anisidinium cation by N—H*C*⋯O*W*2 hydrogen bonds as shown in Fig. 6 ▸. The role of this water mol­ecule can be seen better in Fig. 7 ▸ where mol­ecular stacking along the *b* axis is observed, leaving an empty inter­layer space where O*W*2 mol­ecules are located, ensuring a strong link between organic and inorganic sheets.

There are two types of hydrogen bonds, the first one has nitro­gen as the donor with iodine as an acceptor to form N—H⋯I bonds. The second type has nitro­gen as the donor with oxygen as an acceptor to form N—H⋯O bonds. All these bonds are listed in Table 2 ▸. We have to note that H*W*2*A* is not involved in hydrogen bonding.

## Database survey   

A systematic search procedure in the Cambridge Structural Database (Version 5.36; Groom & Allen, 2014 ▸) based on the *p*-anisidinium cation scheme gives a total of 25 hits. Only two are hybrid compounds: (C_7_H_10_NO)^+^_4_[BiCl_6_]^3−^·Cl^−^·H_2_O (Liu, 2012 ▸) and (C_7_H_10_NO)^+^_2*n*_[Pb_3_I_8_]^2−^_*n*_·2*n*H_2_O (Prakash *et al.*, 2009 ▸).

## Synthesis and crystallization   

The title compound was synthesized by dissolving stoichiometric amounts of bis­muth(III) iodide in *p*-anisidine in a mixture of water and HI. The resulting solution was stirred well and kept at room temperature. Bright-red prismatic crystals were grown by slow evaporation in a couple of weeks. The purity of the synthesized compound was improved by successive recrystallization processes.

## Refinement   

Crystal data, data collection and structure refinement details are summarized in Table 3 ▸. The hydrogen atoms were located in difference Fourier maps. Those attached to carbon were placed in calculated positions (C—H = 0.90–1.00 Å) while those attached to nitro­gen were placed in experimental positions and their coordinates adjusted to give N—H = 0.89 Å. All were included as riding on their parent atoms with isotropic displacement parameters 1.2–1.5 times those of the parent atoms. Hydrogen positions for the water mol­ecules were partly located from a Fourier difference map and partly placed based on geometrical considerations. They are not of sufficient precision to refine the hydrogen-atom positions for the water mol­ecules with angle and distance restraints and they were therefore treated as riding on their parent oxygen atoms.

## Supplementary Material

Crystal structure: contains datablock(s) I. DOI: 10.1107/S2056989015019489/vn2100sup1.cif

Structure factors: contains datablock(s) I. DOI: 10.1107/S2056989015019489/vn2100Isup2.hkl

CCDC reference: 1431306

Additional supporting information:  crystallographic information; 3D view; checkCIF report

## Figures and Tables

**Table 1:** Selected bond lengths ()

BiI2	2.8938(7)	BiI1^ii^	3.1390(8)
BiI3	2.9850(7)	BiI1	3.1842(8)
BiI4^i^	3.0184(8)	BiI4	3.3238(7)

Symmetry codes: (i) 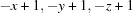; (ii) 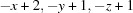.

**Table 2:** Hydrogen-bond geometry (, )

*D*H*A*	*D*H	H*A*	*D* *A*	*D*H*A*
NH*A*I3^iii^	0.89	2.77	3.658(10)	176
NH*B*O*W*1^iv^	0.89	1.97	2.762(12)	147
NH*C*O*W*2	0.89	1.88	2.704(14)	154
O*W*1H*W*1*A*I3	0.85	2.77	3.604(7)	167
O*W*2H*W*2*A*I1^i^	0.85	3.23	3.817(10)	129
O*W*2H*W*2*A*I3	0.85	3.20	3.850(12)	135
O*W*2H*W*2*B*O*W*1^v^	0.85	2.32	2.925(13)	129

Symmetry codes: (i) 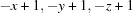; (iii) 
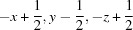
; (iv) 
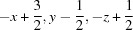
; (v) 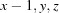.

**Table 3:** Experimental details

Crystal data	
Chemical formula	(C_7_H_10_NO)[BiI_4_]2H_2_O
*M*_r_	876.77
Crystal system, space group	Monoclinic, *P*2_1_/*n*
Temperature (K)	293
*a*, *b*, *c* ()	7.779(2), 12.747(2), 18.252(3)
()	94.97(1)
*V* (^3^)	1803.0(6)
*Z*	4
Radiation type	Mo *K*
(mm^1^)	16.62
Crystal size (mm)	0.6 0.2 0.1

Data collection	
Diffractometer	EnrafNonius CAD-4
Absorption correction	scan (North *et al.*, 1968 ▸)
*T*_min_, *T*_max_	0.014, 0.036
No. of measured, independent and observed [*I* > 2(*I*)] reflections	5050, 3923, 3064
*R*_int_	0.035
(sin /)_max_ (^1^)	0.639

Refinement	
*R*[*F* ^2^ > 2(*F* ^2^)], *wR*(*F* ^2^), *S*	0.035, 0.080, 1.05
No. of reflections	3923
No. of parameters	147
H-atom treatment	H-atom parameters constrained
_max_, _min_ (e ^3^)	1.68, 2.01

Computer programs: *CAD-4 EXPRESS* (EnrafNonius, 1994 ▸), *XCAD4* (Harms Wocadlo, 1995 ▸), *SHELXS97* (Sheldrick, 2008 ▸), *SHELXL2014* (Sheldrick, 2015 ▸), *DIAMOND* (Brandenburg, 2008 ▸) and *publCIF* (Westrip, 2010 ▸).

## supplementary crystallographic information

### Crystal data

**Table:**

(C_7_H_10_NO)[BiI_4_]·2H_2_O	*F*(000) = 1528
*M**_r_* = 876.77	*D*_x_ = 3.230 Mg m^−^^3^
Monoclinic, *P*2_1_/*n*	Mo *K*α radiation, λ = 0.71073 Å
*a* = 7.779 (2) Å	Cell parameters from 25 reflections
*b* = 12.747 (2) Å	θ = 10–15°
*c* = 18.252 (3) Å	µ = 16.62 mm^−^^1^
β = 94.97 (1)°	*T* = 293 K
*V* = 1803.0 (6) Å^3^	Prism, red
*Z* = 4	0.6 × 0.2 × 0.1 mm

### Data collection

**Table:**

Enraf–Nonius CAD-4 diffractometer	3064 reflections with *I* > 2σ(*I*)
Radiation source: fine-focus sealed tube	*R*_int_ = 0.035
Graphite monochromator	θ_max_ = 27.0°, θ_min_ = 2.2°
ω/2θ scans	*h* = −9→1
Absorption correction: ψ scan (North *et al.*, 1968)	*k* = −1→16
*T*_min_ = 0.014, *T*_max_ = 0.036	*l* = −23→23
5050 measured reflections	2 standard reflections every 120 min
3923 independent reflections	intensity decay: 1%

### Refinement

**Table:**

Refinement on *F*^2^	Primary atom site location: structure-invariant direct methods
Least-squares matrix: full	Secondary atom site location: difference Fourier map
*R*[*F*^2^ > 2σ(*F*^2^)] = 0.035	Hydrogen site location: inferred from neighbouring sites
*wR*(*F*^2^) = 0.080	H-atom parameters constrained
*S* = 1.05	*w* = 1/[σ^2^(*F*_o_^2^) + (0.0317*P*)^2^ + 8.0053*P*] where *P* = (*F*_o_^2^ + 2*F*_c_^2^)/3
3923 reflections	(Δ/σ)_max_ = 0.001
147 parameters	Δρ_max_ = 1.68 e Å^−^^3^
0 restraints	Δρ_min_ = −2.01 e Å^−^^3^

### Special details

**Table:**

Geometry. All esds (except the esd in the dihedral angle between two l.s. planes) are estimated using the full covariance matrix. The cell esds are taken into account individually in the estimation of esds in distances, angles and torsion angles; correlations between esds in cell parameters are only used when they are defined by crystal symmetry. An approximate (isotropic) treatment of cell esds is used for estimating esds involving l.s. planes.

### Fractional atomic coordinates and isotropic or equivalent isotropic displacement parameters (Å^2^)

**Table:**

	*x*	*y*	*z*	*U*_iso_*/*U*_eq_	
Bi	0.73731 (4)	0.51922 (2)	0.43418 (2)	0.02320 (9)	
I1	0.93941 (7)	0.62637 (5)	0.57383 (3)	0.03934 (16)	
I2	0.86930 (9)	0.67240 (5)	0.33633 (4)	0.04831 (18)	
I3	0.56183 (8)	0.38519 (5)	0.31619 (3)	0.04102 (16)	
I4	0.58407 (7)	0.35207 (5)	0.55223 (3)	0.03656 (15)	
N	0.2818 (12)	0.0413 (8)	0.2801 (5)	0.059 (2)	
HA	0.1983	0.0059	0.2544	0.071*	
HB	0.3839	0.0176	0.2689	0.071*	
HC	0.2726	0.1092	0.2690	0.071*	
C1	0.2662 (13)	0.0267 (9)	0.3590 (6)	0.048 (3)	
C2	0.1996 (13)	−0.0648 (9)	0.3862 (6)	0.051 (3)	
H2	0.1622	−0.1179	0.3536	0.061*	
C3	0.1880 (13)	−0.0783 (8)	0.4581 (6)	0.047 (2)	
H3	0.1400	−0.1396	0.4750	0.056*	
C4	0.2466 (12)	−0.0020 (7)	0.5077 (6)	0.040 (2)	
C5	0.3162 (13)	0.0920 (8)	0.4821 (6)	0.050 (3)	
H5	0.3546	0.1446	0.5148	0.060*	
C6	0.3261 (13)	0.1043 (8)	0.4071 (6)	0.051 (3)	
H6	0.3734	0.1651	0.3892	0.061*	
C7	0.1791 (15)	−0.1049 (9)	0.6110 (6)	0.062 (3)	
H7A	0.0657	−0.1186	0.5879	0.093*	
H7B	0.1738	−0.0991	0.6632	0.093*	
H7C	0.2550	−0.1613	0.6006	0.093*	
O	0.2425 (9)	−0.0092 (6)	0.5833 (4)	0.0522 (18)	
OW1	0.9289 (10)	0.4148 (6)	0.2103 (4)	0.063 (2)	
HW1A	0.8384	0.3981	0.2307	0.095*	
HW1B	0.9222	0.3883	0.1675	0.095*	
OW2	0.1501 (15)	0.2339 (8)	0.2484 (5)	0.110 (4)	
HW2A	0.1889	0.2855	0.2745	0.166*	
HW2B	0.0502	0.2499	0.2291	0.166*	

### Atomic displacement parameters (Å^2^)

**Table:**

	*U*^11^	*U*^22^	*U*^33^	*U*^12^	*U*^13^	*U*^23^
Bi	0.02239 (16)	0.02297 (16)	0.02394 (14)	−0.00093 (13)	0.00020 (11)	−0.00034 (12)
I1	0.0305 (3)	0.0385 (3)	0.0470 (3)	0.0095 (3)	−0.0082 (2)	−0.0207 (3)
I2	0.0548 (4)	0.0413 (4)	0.0509 (4)	−0.0060 (3)	0.0163 (3)	0.0164 (3)
I3	0.0403 (3)	0.0472 (4)	0.0342 (3)	−0.0063 (3)	−0.0044 (2)	−0.0136 (3)
I4	0.0313 (3)	0.0287 (3)	0.0509 (3)	0.0086 (2)	0.0110 (3)	0.0120 (3)
N	0.056 (6)	0.059 (6)	0.061 (6)	0.007 (5)	0.001 (5)	0.001 (5)
C1	0.034 (5)	0.045 (6)	0.066 (7)	0.008 (5)	0.001 (5)	−0.001 (5)
C2	0.050 (6)	0.040 (6)	0.060 (7)	0.002 (5)	−0.007 (5)	−0.012 (5)
C3	0.046 (6)	0.025 (5)	0.069 (7)	0.000 (4)	0.005 (5)	0.002 (5)
C4	0.027 (5)	0.030 (5)	0.063 (6)	0.005 (4)	−0.001 (4)	0.005 (4)
C5	0.045 (6)	0.028 (5)	0.073 (7)	−0.001 (5)	−0.010 (5)	−0.002 (5)
C6	0.045 (6)	0.034 (6)	0.073 (7)	−0.006 (5)	0.005 (5)	0.010 (5)
C7	0.060 (7)	0.052 (7)	0.071 (8)	−0.008 (6)	−0.010 (6)	0.018 (6)
O	0.051 (4)	0.042 (4)	0.062 (5)	−0.007 (3)	−0.006 (4)	0.000 (4)
OW1	0.063 (5)	0.068 (5)	0.061 (5)	−0.007 (4)	0.019 (4)	0.004 (4)
OW2	0.135 (9)	0.091 (8)	0.098 (8)	0.041 (7)	−0.029 (7)	−0.005 (6)

### Geometric parameters (Å, º)

**Table:**

Bi—I2	2.8938 (7)	C3—C4	1.379 (14)
Bi—I3	2.9850 (7)	C3—H3	0.9300
Bi—I4^i^	3.0184 (8)	C4—O	1.386 (13)
Bi—I1^ii^	3.1390 (8)	C4—C5	1.410 (14)
Bi—I1	3.1842 (8)	C5—C6	1.387 (15)
Bi—I4	3.3238 (7)	C5—H5	0.9300
I1—Bi^ii^	3.1390 (8)	C6—H6	0.9300
I4—Bi^i^	3.0184 (8)	C7—O	1.425 (12)
N—C1	1.468 (14)	C7—H7A	0.9600
N—HA	0.8900	C7—H7B	0.9600
N—HB	0.8900	C7—H7C	0.9600
N—HC	0.8900	OW1—HW1A	0.8518
C1—C6	1.376 (15)	OW1—HW1B	0.8479
C1—C2	1.386 (15)	OW2—HW2A	0.8511
C2—C3	1.336 (14)	OW2—HW2B	0.8499
C2—H2	0.9300		
			
I2—Bi—I3	96.05 (2)	C2—C1—N	121.5 (10)
I2—Bi—I4^i^	91.41 (2)	C3—C2—C1	121.3 (10)
I3—Bi—I4^i^	92.30 (2)	C3—C2—H2	119.4
I2—Bi—I1^ii^	92.39 (2)	C1—C2—H2	119.4
I3—Bi—I1^ii^	86.92 (2)	C2—C3—C4	120.5 (10)
I4^i^—Bi—I1^ii^	176.18 (2)	C2—C3—H3	119.8
I2—Bi—I1	91.58 (2)	C4—C3—H3	119.8
I3—Bi—I1	170.46 (2)	C3—C4—O	124.8 (9)
I4^i^—Bi—I1	93.21 (2)	C3—C4—C5	119.8 (10)
I1^ii^—Bi—I1	87.07 (2)	O—C4—C5	115.4 (9)
I2—Bi—I4	177.35 (2)	C6—C5—C4	118.6 (10)
I3—Bi—I4	86.19 (2)	C6—C5—H5	120.7
I4^i^—Bi—I4	87.08 (2)	C4—C5—H5	120.7
I1^ii^—Bi—I4	89.14 (2)	C1—C6—C5	120.3 (10)
I1—Bi—I4	86.33 (2)	C1—C6—H6	119.9
Bi^ii^—I1—Bi	92.93 (2)	C5—C6—H6	119.9
Bi^i^—I4—Bi	92.92 (2)	O—C7—H7A	109.5
C1—N—HA	109.5	O—C7—H7B	109.5
C1—N—HB	109.5	H7A—C7—H7B	109.5
HA—N—HB	109.5	O—C7—H7C	109.5
C1—N—HC	109.5	H7A—C7—H7C	109.5
HA—N—HC	109.5	H7B—C7—H7C	109.5
HB—N—HC	109.5	C4—O—C7	116.7 (8)
C6—C1—C2	119.6 (11)	HW1A—OW1—HW1B	108.5
C6—C1—N	118.9 (10)	HW2A—OW2—HW2B	108.4

Symmetry codes: (i) −*x*+1, −*y*+1, −*z*+1; (ii) −*x*+2, −*y*+1, −*z*+1.

### Hydrogen-bond geometry (Å, º)

**Table:**

*D*—H···*A*	*D*—H	H···*A*	*D*···*A*	*D*—H···*A*
N—H*A*···I3^iii^	0.89	2.77	3.658 (10)	176
N—H*B*···O*W*1^iv^	0.89	1.97	2.762 (12)	147
N—H*C*···O*W*2	0.89	1.88	2.704 (14)	154
O*W*1—H*W*1*A*···I3	0.85	2.77	3.604 (7)	167
O*W*2—H*W*2*A*···I1^i^	0.85	3.23	3.817 (10)	129
O*W*2—H*W*2*A*···I3	0.85	3.20	3.850 (12)	135
O*W*2—H*W*2*B*···O*W*1^v^	0.85	2.32	2.925 (13)	129

Symmetry codes: (i) −*x*+1, −*y*+1, −*z*+1; (iii) −*x*+1/2, *y*−1/2, −*z*+1/2; (iv) −*x*+3/2, *y*−1/2, −*z*+1/2; (v) *x*−1, *y*, *z*.
